# Coffeehouses as Symbolic Spaces: Materialism and Experiential Motivations in Coffeehouse Selection

**DOI:** 10.3390/foods15203585

**Published:** 2026-10-09

**Authors:** Ceyhun Uçuk, Uzeyir Kement, Riccardo Migliavada, Nilufer Sahin, Onurcan Arman, Luisa Torri, Charles Spence

**Affiliations:** 1 Department of Experimental Psychology, University of Oxford, Life and Mind Building, South Parks Road, Oxford OX1 3EL, UK; ceyhun.ucuk@psy.ox.ac.uk or ceyhunucuk@gantep.edu.tr; 2 Department of Gastronomy and Culinary Arts, Faculty of Tourism, Gaziantep University, 27100 Gaziantep, Türkiye; 3 Department of Gastronomy and Culinary Arts, Faculty of Tourism, Ordu University, 52200 Ordu, Türkiye; uzeyirkement@gmail.com; 4 University of Gastronomic Sciences, Piazza Vittorio Emanuele II 9, 12042 Pollenzo, Italy; r.migliavada@unisg.it (R.M.); l.torri@unisg.it (L.T.); 5 Department of Gastronomy and Culinary Arts, Faculty of Tourism, Nevsehir Haci Bektas Veli University, 50100 Nevsehir, Türkiye; nilufer.percin@nevsehir.edu.tr; 6 School of Exercise and Nutrition Sciences, Faculty of Health, Deakin University, Melbourne 3125, Australia; onurcanarman132@gmail.com

**Keywords:** cross-cultural analysis, consumer behavior, coffee, coffeehouse, materialism, PLS-SEM

## Abstract

The demand for coffeehouses has been growing globally; however, little is known about the psychological factors associated with individuals’ coffeehouse selection. Drawing on a value–motivation–intention framework, this study examines how materialism–coffeehouse as defining success (M-CDS), as an underlying value orientation, relates to snob, bandwagon, hedonic consumption tendency (HCT), and eudaimonic orientations, and how these orientations are associated with customers’ willingness to select coffeehouses. Survey data collected from Türkiye (n = 286) and Italy (n = 315) were analyzed using PLS-SEM. The findings showed that M-CDS was positively associated with all four psychological orientations, each of which was positively associated with willingness to select coffeehouses. Significant indirect effects were also observed through all four orientations, with the eudaimonic pathway showing the largest specific indirect effect. Multi-group analysis identified several between-country differences: the relationship between eudaimonic motivation and willingness was stronger among Italian participants, whereas the relationship between HCT and willingness was stronger among Turkish participants. These findings indicate that coffeehouse selection is associated not only with functional considerations but also with consumers’ value orientations and social and experiential motivations. The study extends previous research by incorporating the eudaimonic pathway and examining these relationships across Turkish and Italian samples, while offering practical insights for coffeehouse managers operating across different markets.

## 1. Introduction

Coffee is one of the most demanded and consumed products in the world [1]. Over the years, coffee consumption patterns and consumers’ perspectives on coffee have shifted towards a more specialty-oriented approach [2]. In this context, examining consumers’ decision-making mechanisms in selecting the venue where they choose to drink coffee has received increasing attention [3]. Samoggia and Riedel [1] suggest that important determinants of coffee consumption may be clustered into several categories, such as personal choices, product and economic features, and socio-demographic factors. There are numerous stimuli in coffeehouses that influence individuals’ coffeehouse selection, including the aroma, flavor, and appearance of coffee; the ambiance, music, and design of the space; and interactions with other customers and staff [4,5]. Beyond these environmental characteristics, consumers’ coffeehouse choices may also be associated with underlying personal values and consumption-related psychological orientations [6]. However, the relationships through which such value orientations are associated with consumers’ social and experiential consumption orientations and, ultimately, their willingness to select coffeehouses remain underexplored. Therefore, further research is needed in this regard.

According to the International Coffee Organization (ICO), coffee consumption in the world increased in 2022 as a result of the accumulated demand after COVID-19 [7]. Because global coffee consumption is growing faster than production, it is important to understand which countries and cultures are driving that increase [7]. Türkiye has a cultural and traditional consumption habit of Turkish coffee [8]. Similarly, Italy also has a deep cultural and traditional consumption habit of espresso coffee [9]. These deep-rooted but distinct traditions make Türkiye and Italy a particularly informative pairing for examining coffeehouse consumption from a cross-cultural perspective.

According to the ICO [7], global coffee consumption has risen steadily over the past few years, increasing from approximately 167 million bags in 2020/2021 to 177 million bags in 2022/2023. This increase has led to coffee being considered not only as a functional beverage but also as a consumption practice carrying social, cultural, and experiential meaning. Indeed, coffee consumption is transforming into a symbolic experiential space where individuals can reflect on their lifestyles, social identities, and consumption preferences [10]. Therefore, individuals’ coffeehouse preferences are shaped not only by the product itself but also by psychological and social motivations such as the pursuit of pleasure, social visibility, a sense of belonging, and experiential expectations [11,12].

In this context, Türkiye and Italy offer two remarkable cultural examples due to their deep-rooted coffee traditions. In Türkiye, Turkish coffee consumption has historically been seen as an important part of social interaction, hospitality, and cultural sharing [8]. Similarly, in Italy, coffee culture plays a central role in everyday life, with coffee consumption being deeply embedded in daily social routines (e.g., espresso, cappuccino, and caffè macchiato) [9]. However, it is thought that the way coffee is consumed and the meaning attributed to coffeehouses may differ in the two cultures. While the consumption of coffee in Italy is more often seen as a fast, routine part of daily life [9,13], in Türkiye, the coffeehouse experience tends to be associated with more extended socialization, experiential sharing, and social interaction [14]. Therefore, it is considered that these differences in terms of how coffee is culturally interpreted may also differentiate the psychological motivations and consumption tendencies that influence individuals’ coffeehouse preferences.

Understanding how materialistic tendencies shape individuals’ social and experiential consumption motivations can help explain the processes underlying coffeehouse preference. A value–motivation–intention perspective provides a useful conceptual framework for examining these relationships by considering how underlying consumer values may be associated with more proximal psychological and motivational orientations and, subsequently, behavioral intentions [15].

Materialism is particularly relevant within this framework because it has been conceptualized as a consumer value reflecting the importance individuals attach to possessions and their acquisition. One dimension of this value orientation is possession-defined success, whereby possessions are used as indicators for evaluating achievement and success [16]. In consumption contexts, such a value orientation may be associated with different psychological orientations through which consumers attach social and experiential significance to their choices. Previous research on coffee consumption has shown that materialism is positively associated with snob, bandwagon, and hedonic consumption tendencies [17]. Consumption experiences, however, may extend beyond social and hedonic considerations and include eudaimonic aspects associated with meaning, personal fulfillment, and personal growth [18]. Accordingly, incorporating eudaimonic motivation provides an additional pathway for examining how materialistic value orientations may be associated with coffeehouse selection.

Building on this reasoning, the present study organizes its conceptual model according to a value–motivation–intention framework. Materialism—specifically, its contextualized expression as defining success through coffeehouse-related consumption (M-CDS)—represents the underlying value orientation. The snob effect, bandwagon effect, hedonic consumption tendency (HCT), and eudaimonic motivation represent distinct psychological orientations through which M-CDS may be associated with coffeehouse selection, while customers’ willingness to select coffeehouses represents the behavioral intention. Accordingly, this study aims to examine, from a cross-cultural perspective, the relationships between M-CDS and individuals’ HCT, eudaimonic, bandwagon, and snob orientations; the relationships between these orientations and customers’ willingness to select coffeehouses; and whether these relationships differ between the Turkish and Italian samples. Accordingly, the research sought answers to the following questions:

*RQ1:* How does M-CDS relate to individuals’ HCT, eudaimonic, bandwagon, and snob orientations?
*RQ2:* How do HCT, eudaimonic, bandwagon, and snob orientations relate to individuals’ willingness to select coffeehouses?
*RQ3:* Do HCT, eudaimonic, bandwagon, and snob orientations mediate the relationships between M-CDS and customers’ willingness to select coffeehouses?
*RQ4:* Do the relationships among M-CDS, psychological orientations, and willingness to select coffeehouses differ between individuals in Türkiye and Italy?

Niyet and Taşpınar [17] established that materialistic individuals display stronger snob, HCT, and bandwagon tendencies in coffee consumption—corresponding to H_1a_, H_1b_, and H_1c_ of the present study. What remains unexamined is whether materialism also relates to eudaimonic motivation, and whether these relationships hold uniformly across cultural contexts with markedly different coffeehouse traditions. The present study extends [17] along both dimensions: by incorporating the eudaimonic pathway, and by testing the full structural model separately across two culturally distinct samples (Türkiye and Italy) via multi-group analysis.

### 1.1. Value–Motivation–Intention Framework

Consumer values can provide an underlying basis for understanding how individuals make consumption-related decisions. Rather than being directly translated into behavioral intentions, values may operate through more proximal motivational mechanisms that give direction to consumer choice. Consistent with this logic, previous consumer research has used a value–motivation–intention model to examine how consumption values relate to behavioral intentions through motivational mechanisms [15]. Accordingly, a value–motivation–intention logic provides a useful conceptual basis for examining how an underlying value orientation may be associated with consumption-related psychological orientations and, subsequently, behavioral intention.

Materialism is particularly relevant to this conceptualization because it has been defined as a consumer value concerning the importance individuals attach to possessions and their acquisition. Importantly, one dimension of this value orientation is possession-defined success, whereby individuals evaluate their own and others’ success on the basis of possessions [16]. In consumption settings, such a value orientation may be associated with different psychological orientations through which consumers attach social and experiential significance to their choices. In the context of coffee consumption, previous research has shown positive relationships between materialism and snob, bandwagon, and hedonic consumption tendencies [17]. These orientations reflect different aspects of consumption, including differentiation from others, conformity with socially-valued consumption patterns, and the pursuit of pleasurable experiences.

Consumption experiences, however, are not limited to social and hedonic considerations. They may also involve eudaimonic aspects associated with meaning, personal fulfillment, and personal growth. Research on consumers’ lived food experiences has specifically conceptualized food well-being as an eudaimonic experience involving such elements [18]. This perspective provides a basis for considering eudaimonic motivation alongside the social and hedonic orientations previously examined in relation to materialism and coffee consumption. Accordingly, incorporating eudaimonic motivation provides an additional pathway for examining how materialistic value orientations may be associated with coffeehouse selection.

Building on this reasoning, the present study organizes its conceptual model according to a value–motivation–intention logic. Materialism—more specifically, its contextualized expression as defining success through coffeehouse-related consumption (M-CDS)—represents the underlying value orientation. The snob effect, bandwagon effect, hedonic consumption tendency (HCT), and eudaimonic motivation represent distinct psychological orientations through which M-CDS may be associated with coffeehouse selection, while customers’ willingness to select coffeehouses represents the behavioral intention. Accordingly, the study examines the relationships between M-CDS and these four psychological orientations and, in turn, the relationships between these orientations and customers’ willingness to select coffeehouses. In this way, the study extends previous research on materialism and coffee consumption [17] by incorporating an eudaimonic pathway and examining the proposed relationships across Turkish and Italian samples.

### 1.2. Cultural Differences in Coffeehouse Selection Between Türkiye and Italy

Socio-cultural traditions and life practices play a significant role in shaping individuals’ food and beverage consumption habits [19]. In this context, coffee, both in Türkiye and Italy, is considered more than just a beverage; it is a cultural consumption element integrated into social life. Turkish coffee culture is historically associated with hospitality, social sharing, and social interaction, while coffeehouses have functioned as important social gathering places since the Ottoman period [20]. Traditional coffeehouses are not only places where coffee is consumed; they also stand out as social spaces where social interaction, conversation, and cultural sharing take place [21].

Similarly, in Italy, coffee is considered an important component of the culture of daily life [22]. Italian coffeehouses are considered social living spaces, particularly shaped around an espresso-based consumption culture [9,22]. However, while coffee consumption in Italy is mostly a fast-paced and repetitive practice integrated into daily routines, in Türkiye, coffee consumption gains meaning more in the context of social interaction, hospitality, and experiential sharing [20]. Therefore, it is thought that these differences in how coffee is culturally understood may also differentiate individuals’ psychological motivations, social expectations, and consumption tendencies regarding their choice of coffeehouses.

### 1.3. Literature Review and Hypothesis Development

Materialism is a consumer value orientation reflecting the importance and value that individuals place on material possessions [23]. It is also closely linked to status-oriented consumption behaviors. Individuals with higher materialistic tendencies are more inclined to gravitate towards privileged, prestigious, and high-status products to demonstrate their social status. Indeed, Tascioglu et al. [24] revealed that materialism affects individuals’ status motivation and that the aspect related to the pursuit of uniqueness strengthens snob tendencies. Similarly, Mainolfi [25], in his study on Chinese consumers, determined that materialistic tendencies increase individuals’ willingness to purchase luxury products through snob and bandwagon effects.

It is also noted that materialistic individuals gravitate towards popular or prestigious products in order to gain acceptance from their social environment and strengthen their status [26]. Rhee and Johnson [27] showed that individuals with high levels of materialistic tendencies are more likely to prefer products that are popular among their peers. Similarly, Dinh and Lee [28] state that materialistic individuals are more inclined to follow consumption trends promoted by social media influencers. This indicates that materialism is related not only to individual desire for possession but also to social approval and group belonging.

Materialism is also associated with pleasure-oriented consumption tendencies. Kim et al. [29] revealed that materialistic individuals interact more with sponsored content due to the pleasure they derive from consumption. Although meta-analytic evidence generally indicates a negative association between materialism and subjective well-being [30], this relationship appears to depend on how materialism is conceptualized and on the consumption context. In experiential and symbolic consumption settings, materialistic individuals may derive meaning, belonging, and self-expression from consumption experiences [31], suggesting that materialism may positively relate to eudaimonic motivations in such contexts. Accordingly, within the value–motivation–intention framework adopted in this study, materialism is expected to be associated with both the social and experiential psychological orientations underlying coffeehouse selection. Drawing on these findings, the following hypotheses were formulated:

**H_1a_.**  *Materialism has a positive effect on the snob effect*.

**H_1b_.**  *Materialism has a positive effect on the bandwagon effect*.

**H_1c_.**  *Materialism has a positive effect on the hedonic consumption tendency*.

**H_1d_.**  *Materialism has a positive effect on the eudaimonic effect*.

Researchers have investigated the drivers and motivations that influence individuals’ coffeehouse selections to better understand consumer preferences [1]. For example, Niyet and Taşpınar [17] examined individuals’ consumption tendencies regarding coffee. The study found that materialistic individuals were more likely to display snob, HCT, and bandwagon purchasing behaviors. Similarly, Kim and Jang [32] explored the roles of price and prestige in consumer decision-making, suggesting that snob and bandwagon effects were related to individuals’ perceptions of the quality of coffeehouses. Ersoy et al. [33] explored the role of social media influence on coffeehouse selection, suggesting that coffeehouses that had higher social media engagement with consumers attracted more visitors. This supports the idea that social validation is a crucial factor in consumer choices [34]. Sensory experiences also have a key role in shaping consumer behavior, especially in the context of coffee consumption. Carvalho and Spence [35] investigated how multisensory cues, such as the color, cup design, and overall presentation of coffee, influenced consumer enjoyment. The results suggested that these sensory components may enhance the overall hedonic experience of coffee consumption, thus affecting individuals’ coffeehouse selections [35]. Tumanan and Lansangan [36] suggest that coffee consumption may not only be associated with habits but also has socio-cultural and affective meanings. Moreover, Smith Maguire and Hu [37] suggest that coffee consumption in consumer-preferred coffeehouses may demonstrate their social status as being ‘modern’, thus enhancing their sense of fulfilment. Taken together, these findings indicate that coffeehouse selection may be associated with social, hedonic, and experiential psychological orientations, consistent with the motivational component of the value–motivation–intention framework adopted in this study. In line with these findings, the following hypotheses were developed:

**H_2_.**  *The snob effect has a positive effect on willingness to select coffeehouses*.

**H_3_.**  *The bandwagon effect has a positive effect on willingness to select coffeehouses*.

**H_4_.**  *The hedonic consumption tendency has a positive effect on willingness to select coffeehouses*.

**H_5_.**  *The eudaimonic effect has a positive effect on willingness to select coffeehouses*.

Beyond these direct relationships, the value–motivation–intention framework also provides a basis for considering indirect relationships, whereby underlying consumer values may be associated with behavioral intentions through more proximal psychological and motivational orientations. Accordingly, M-CDS, as the underlying value orientation in the present study, is expected to be associated with customers’ willingness to select coffeehouses through these psychological orientations. While Mainolfi [25] established that bandwagon-type luxury consumption serves as a mediating mechanism linking materialism to luxury purchase intentions, Niyet and Taşpınar [17] revealed that materialism’s association extends beyond the bandwagon effect to include snob and hedonic consumption tendencies as well. Considering that HCT and eudaimonic motivations may connect consumers’ value orientations with experiential consumption decisions [38], such indirect pathways are also likely in the coffeehouse context. Therefore, the relationship between M-CDS and customers’ willingness to select coffeehouses is expected to be mediated by the snob, bandwagon, HCT, and eudaimonic orientations, and the following hypothesis is proposed:

**H_6_.**  *The bandwagon effect (H_6a_), HCT (H_6b_), snob effect (H_6c_), and eudaimonic effect (H_6d_) mediate the relationship between materialism and customers’ willingness to select coffeehouses*.

Considering a popular culture product like coffee, national or regional cultural differences may be associated with variations in these relationships. Therefore, this research compares two countries: Italy, where coffee consumption is high and which is the origin of many contemporary coffee preparation styles such as espresso, and Türkiye, which has a centuries-old coffeehouse tradition and where coffee consumption practices have evolved considerably in recent years. In Italy, coffee is largely integrated into fast-paced daily routines centered around espresso [9], whereas in Türkiye, the coffeehouse experience is more strongly associated with extended socialization, hospitality, and experiential sharing [14]. Moreover, since socio-cultural traditions shape both consumption motivations and the symbolic meanings attributed to consumption spaces [21,39], the strength of the hypothesized relationships may differ across the two cultural contexts.

**H_7_.**  *The hypothesized relationships differ between Turkish and Italian participants*.

**Figure 1:**
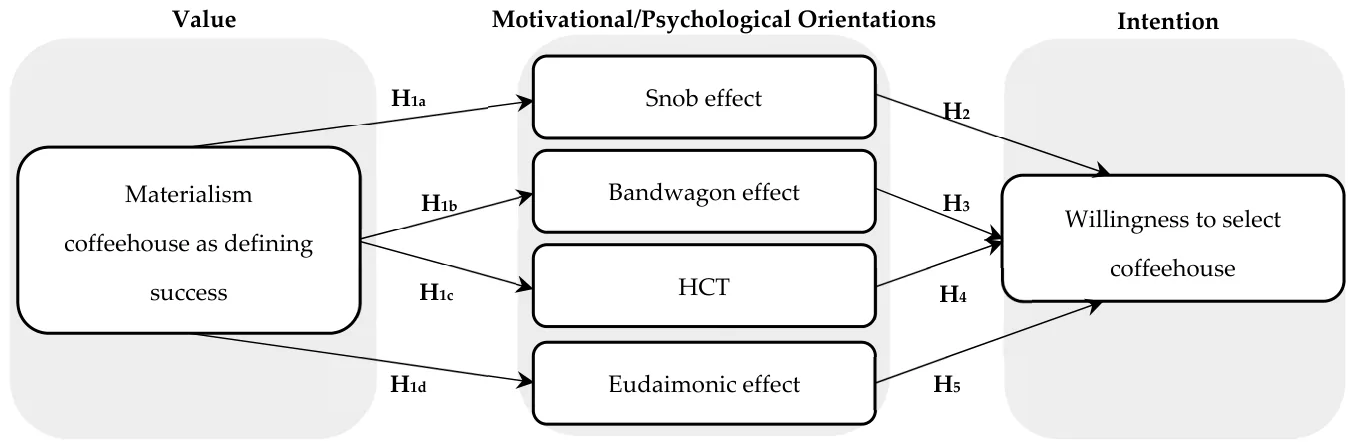
Research model. HCT = Hedonic consumption tendency.

Figure 1 presents the conceptual model of the research. In the model, M-CDS represents the underlying value orientation, while HCT, eudaimonic, bandwagon, and snob orientations represent distinct psychological and motivational orientations associated with customers’ willingness to select coffeehouses. Accordingly, the model examines the relationships between M-CDS and these four orientations and, in turn, their relationships with customers’ willingness to select coffeehouses.

## 2. Materials and Methods

### 2.1. Population and Measures

Participants were individuals aged 18 years or older who patronized coffeehouses in either Türkiye or Italy and were recruited using convenience sampling. The survey was administered online through QualtricsXM between 25 December 2022 and 6 June 2023. Data collection was concluded when 601 completed responses had been obtained, comprising 286 participants from Türkiye and 315 from Italy. Incomplete questionnaires were not included in the analytical dataset. Sample size adequacy was assessed a priori using G*Power 3.1.9.7 [40], based on a four-predictor multiple regression model (*f*^2^ = 0.05, α = 0.05, power = 0.95), which indicated a minimum required sample of 377 participants; thus, the final analytical sample exceeded the minimum requirement. In addition, sensitivity analyses were conducted separately for the Turkish (n = 286) and Italian (n = 315) samples to assess statistical power at the group level. With α = 0.05, power = 0.95, and four predictors, the minimum detectable effect sizes were *f*^2^ = 0.066 for Türkiye and *f*^2^ = 0.060 for Italy, indicating sensitivity to small-to-moderate effects within each country sample. Because the original measurement items were in English, a translation–back-translation procedure was used to develop the Turkish and Italian versions. Academic specialists in tourism and consumer behavior reviewed the translated questionnaires for wording clarity and cultural appropriateness, and a pilot test was conducted before minor linguistic refinements were made to the final questionnaire. Participation was entirely voluntary, written informed consent was obtained from all participants, and ethical approval was obtained from the relevant institutional committee. Additional details on the administration and reporting of the online survey are provided in the completed CHERRIES checklist (Supplementary Table S1).

The questionnaire consists of two parts. In the first part, items measuring the demographic characteristics of the participants are included. In the second part, four items were used to measure materialism [41,42], five items to measure bandwagon effect [41,43], four items to measure the snob effect [41,44], four items to measure HCT [45], four items to measure eudaimonic effect [38], and two items were used to measure customers’ willingness to select coffeehouses [46]. All scales were adapted to the sample for use in this research context. The items were presented to the participants using a five-point Likert scale.

Regarding age, the largest group in Türkiye was participants aged 25–34 years (51.7%), whereas in Italy it was those aged 65 years and older (25.4%) (Table 1). The Turkish sample was predominantly female (64.0%), while the Italian sample was predominantly male (69.2%). Single participants constituted the majority in Türkiye (73.8%), whereas married participants were slightly more prevalent in Italy (52.7%). Regarding education, bachelor’s degree holders represented the largest group in Türkiye (31.1%), while master’s degree holders were the largest group in Italy (37.5%). Middle income was the most frequently reported self-perceived income level in both Türkiye (60.5%) and Italy (73.7%). Finally, the most common frequency of coffeehouse visits was rarely in Türkiye (37.1%) and 1–2 times per week in Italy (30.2%). These descriptive differences indicate that the two national samples were not demographically equivalent, which should be considered when interpreting subsequent cross-country comparisons.

### 2.2. Analysis

Data were analyzed using structural equation modeling (SEM) in SmartPLS 4, chosen because it does not require the assumption of normally distributed data [47] and because its multi-group analysis (MGA) capability suited a dataset drawn from two countries. The procedure followed five steps: assessing the measurement model for scale validity and reliability, estimating the model’s predictive power; testing the hypotheses—including the mediation effect—through the structural model; and finally running a multi-group analysis to compare the two country samples. To complement the PLS-SEM results, importance–performance map analysis (IPMA) was conducted to identify constructs that are relatively important in explaining customers’ willingness to select coffeehouses but exhibit comparatively low performance, thereby highlighting potential priority areas for improvement [48,49]. Given that responses were self-reported and gathered from a single source within a cross-sectional design, common method bias (CMB) was a potential concern. Harman’s single-factor test initially showed that the first factor explained 36.90% of the total variance, below the conventional 50% threshold [50]. Given the limitations of relying solely on Harman’s test, CMB was additionally assessed using the full collinearity VIF approach. All full collinearity VIF values were below the recommended threshold of 3.3, providing additional evidence that CMB was unlikely to substantially affect the results [51].

## 3. Results

### 3.1. Measurement Model Assessment

Table 2 reports construct reliability and validity. A confirmatory factor analysis of outer loadings led to dropping “material4” for falling below the 0.50 cut-off [52]; the rest loaded above this level, supporting item reliability. For reliability more broadly, Cronbach’s alpha, rho_a, and composite reliability (rho_c) were checked. Materialism (α = 0.630) and willingness (α = 0.476) fell short of the usual 0.70 alpha benchmark, but this is expected given alpha’s tendency to understate reliability for short scales [47]; PLS-SEM guidance therefore favors rho_c as the primary indicator [47], and here every construct cleared 0.70 [53], with AVE values above 0.50 confirming convergent validity [54].

Discriminant validity was checked via the Fornell–Larcker criterion (Table 3). Each construct’s AVE square root exceeded its correlations with other constructs [54]. Model fit was also acceptable—SRMR came in at 0.073, under the 0.080 benchmark [55]—while NFI, which ranges from 0 to 1.00 [56], reached 0.771, below the conventional 0.90 threshold, though fit indices adapted from covariance-based SEM have limited diagnostic value in variance-based PLS-SEM and are reported here for transparency rather than as a model acceptance criterion [52].

### 3.2. Structural Model Assessment

InnerVIF (Inner variance inflation factor) was calculated to determine whether the scales have multicollinearity problems. Accordingly, the InnerVIF values of all scales remained below the more conservative 3.3 threshold [57] (the highest value, 3.025 for H5, exceeds the stricter 3.00 rule of thumb but remains well under 3.3). Second, the determination coefficient (R^2^) was calculated. The determination rate of the independent variables of materialism is, respectively: for the bandwagon effect—0.522, for the eudaimonic effect—0.511, for the HCT—0.424, and for the snob effect—0.068. The determination rate of customers’ willingness to select coffeehouses was 0.349. The effect sizes (*f*^2^) were also assessed (see Table 4). According to Cohen [58], *f*^2^ values of 0.02, 0.15, and 0.35 indicate small, medium, and large effects, respectively. Materialism had a large effect on the bandwagon (*f*^2^ = 1.094), eudaimonic (*f*^2^ = 1.047), and HCT (*f*^2^ = 0.736), and a small effect on the snob effect (*f*^2^ = 0.073). The effects of the eudaimonic (*f*^2^ = 0.045), HCT (*f*^2^ = 0.023), snob (*f*^2^ = 0.019), and bandwagon effects (*f*^2^ = 0.018) on customers’ willingness to select coffeehouses were small.

### 3.3. Predictive Model Assessment

The PLS predict assessment showed positive Q^2^_predict_ values for all indicators, indicating that the PLS-SEM model has predictive relevance relative to the naïve benchmark [59,60]. However, comparison of the PLS-SEM mean absolute error (MAE) values with those of the linear model (LM) benchmark produced mixed results. PLS-SEM yielded lower MAE values than the LM for three of the five bandwagon indicators and three of the four eudaimonic indicators, whereas it outperformed the LM for only one of the four hedonic indicators and for none of the snob or willingness indicators. Thus, the model does not demonstrate a consistent predictive advantage over the LM benchmark. This interpretation is further supported by the cross-validated predictive ability test (CVPAT) [61], for which the overall average loss difference between PLS-SEM and the LM was positive but not statistically significant (0.007, *p* = 0.065). Accordingly, although the model exhibits predictive relevance, the results do not provide evidence of an overall out-of-sample predictive advantage over the LM benchmar (see Table 5).

**Figure 2:**
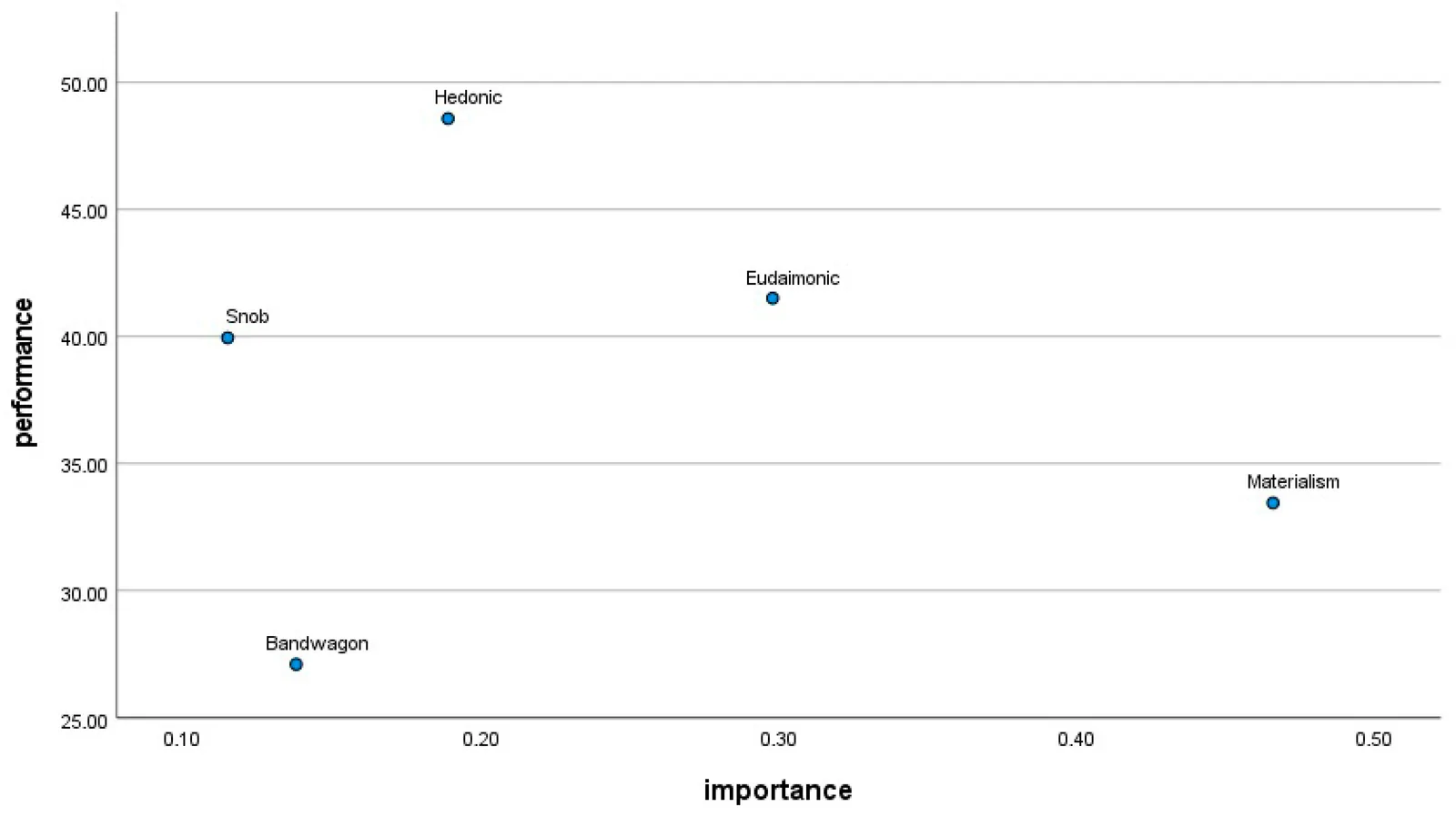
IPMA results. Source. Created by authors, Bwagon = Bandwagon effect.

The IPMA results (see Figure 2) were divided into four quadrants using the mean importance (0.241) and mean performance (38.12) values as reference points. Materialism was positioned in the high-importance/low-performance quadrant, exhibiting the highest importance for customers’ willingness to select coffeehouses (0.466) but comparatively low performance (33.45). This positioning identifies materialism as a priority area for managerial attention. Eudaimonic motivation was located in the high-importance/high-performance quadrant, whereas hedonic consumption tendency and snob motivation were positioned in the low-importance/high-performance quadrant. Bandwagon motivation was located in the low-importance/low-performance quadrant.

### 3.4. Structural Equation Model Results

**Figure 3:**
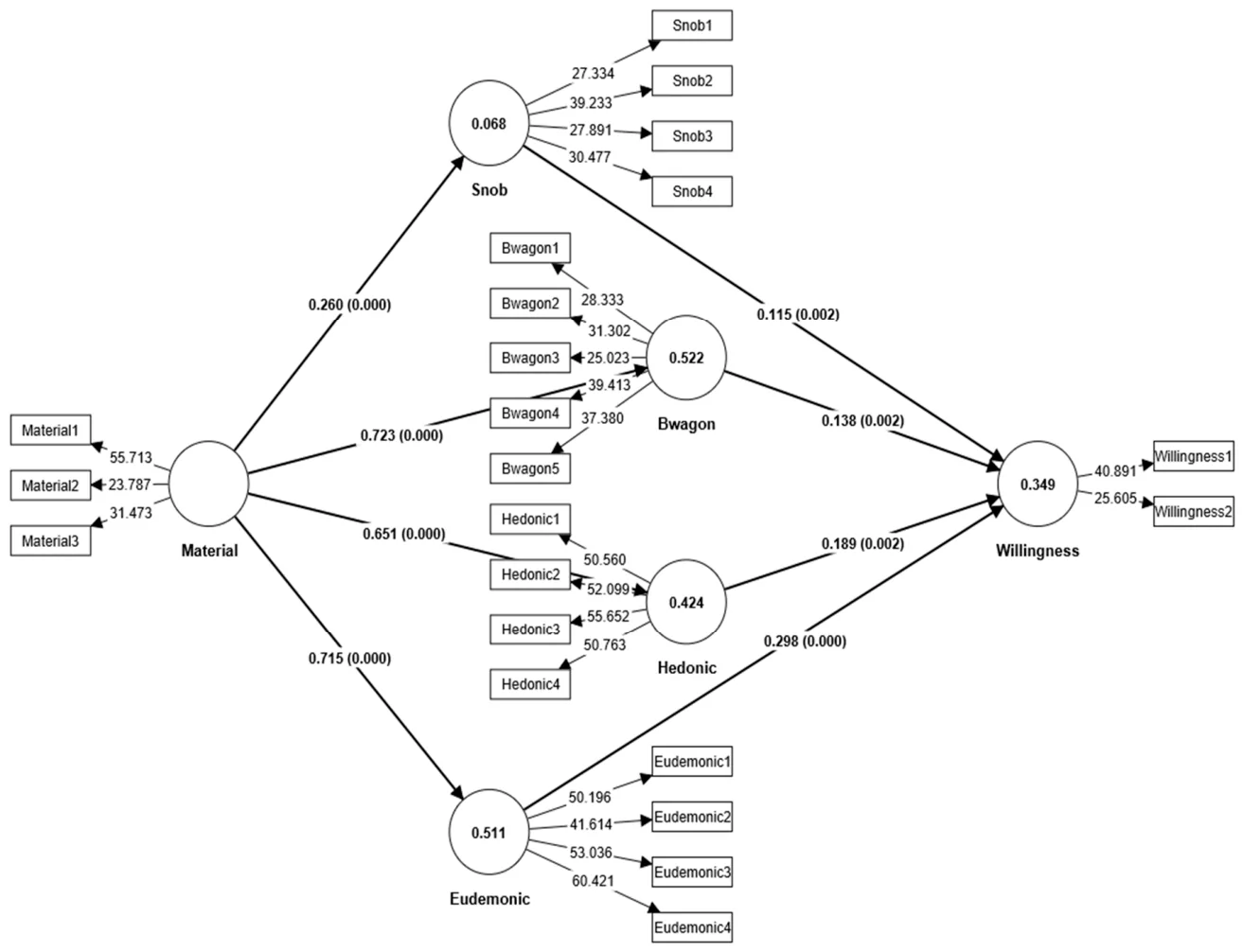
Structural equation modeling results. Source. Created by authors, Willingness = Customers’ willingness to select coffeehouses, Hedonic = Hedonic consumption tendency.

The structural equation modeling results are reported in Table 4 and Figure 3. The significance of the structural path coefficients and indirect effects was assessed using bootstrapping with 5000 resamples and percentile-based confidence intervals. Materialism has a significant and positive effect on the snob effect (β = 0.260, t = 6.192, *p* = 0.000), bandwagon effect (β = 0.723, t = 30.991, *p* = 0.000), *HCT* (β = 0.651, t = 26.562, *p* = 0.000), and eudaimonic effect (β = 0.715, t = 33.329, *p* = 0.000), thus supporting H_1a_, H_1b_, H_1c_, and H_1d_. The snob effect (β = 0.115, t = 3.114, *p* = 0.002), bandwagon effect (β = 0.138, t = 3.085, *p* = 0.002), *HCT* (β = 0.189, t = 3.088, *p* = 0.002), and eudaimonic effect (β = 0.298, t = 4.298, *p* = 0.000) all had a significant and positive effect on customers’ willingness to select coffeehouses, hence supporting hypotheses H_2_, H_3_, H_4_, and H_5_.

The mediating roles of the snob, bandwagon, HCT, and eudaimonic effects in the relationship between materialism and customers’ willingness to select coffeehouses were also evaluated (see Table 6). Following Zhao et al. [62], mediation was assessed based on the significance of the indirect effects. The results showed significant positive indirect effects through the snob effect (β = 0.030, t = 2.701, *p* = 0.007), bandwagon effect (β = 0.100, t = 3.048, *p* = 0.002), HCT (β = 0.123, t = 3.022, *p* = 0.003), and eudaimonic effect (β = 0.213, t = 4.228, *p* = 0.000). Thus, the results provide evidence of mediation through all four mechanisms, supporting H_6a_, H_6b_, H_6c_, and H_6d_.

### 3.5. Country-Specific Model Assessment

To further assess the model within each cultural context, the measurement and structural models were estimated separately for the Turkish and Italian samples. Regarding the measurement models, all retained indicators had outer loadings above 0.50 [47]. Rho_c values exceeded 0.70 and AVE values exceeded 0.50 for all constructs in both samples, supporting composite reliability and convergent validity. Discriminant validity was also established for both country-specific models according to the Fornell–Larcker criterion [54].

The country-specific structural models showed differences in explanatory power. In the Turkish sample, M-CDS explained 53.9% of the variance in the bandwagon effect, 60.9% in the eudaimonic effect, 50.7% in HCT, and 1.6% in the snob effect. The four psychological orientations jointly explained 33.7% of the variance in willingness to select coffeehouses. In the Italian sample, the corresponding R^2^ values were 0.465 for the bandwagon effect, 0.407 for the eudaimonic effect, 0.349 for HCT, and 0.119 for the snob effect, while 35.4% of the variance in willingness to select coffeehouses was explained.

The country-specific path analyses further revealed distinct patterns. In Türkiye, M-CDS was significantly associated with the bandwagon effect (β = 0.734, *p* < 0.001), HCT (β = 0.712, *p* < 0.001), and the eudaimonic effect (β = 0.781, *p* < 0.001), whereas its relationship with the snob effect did not reach statistical significance (β = 0.127, *p* = 0.054). Regarding willingness to select coffeehouses, the snob effect (β = 0.157, *p* = 0.007) and HCT (β = 0.341, *p* = 0.001) were significant, whereas the bandwagon (β = 0.080, *p* = 0.253) and eudaimonic effects (β = 0.157, *p* = 0.181) were not. In Italy, M-CDS was significantly associated with the snob (β = 0.345, *p* < 0.001), bandwagon (β = 0.682, *p* < 0.001), HCT (β = 0.590, *p* < 0.001), and eudaimonic effects (β = 0.638, *p* < 0.001). However, only the eudaimonic effect was significantly associated with willingness to select coffeehouses (β = 0.446, *p* < 0.001); the snob (β = 0.026, *p* = 0.604), bandwagon (β = 0.118, *p* = 0.055), and HCT effects (β = 0.088, *p* = 0.251) were not statistically significant. Detailed country-specific structural models are presented in Figures S1 and S2 in the Supplementary Materials.

### 3.6. Multi-Group Analysis

To compare the structural relationships of Turkish and Italian participants, MGA was applied following an MICOM check for measurement invariance [63,64]. This confirmed configural invariance (identical models and settings across groups) and compositional invariance (via permutation testing), while composite means and variances differed slightly. As partial invariance was thus achieved, comparing path coefficients across the two groups through MGA was deemed justified.

Multiple group analysis was conducted to examine whether the structural relationships differed between Italian and Turkish participants (see Table 7). The results revealed significant between-country differences in several paths. Specifically, the effect of the *eudaimonic effect* on *willingness to select coffeehouses* was stronger among Italian participants, whereas the effect of the *HCT* on *willingness to select coffeehouses* was stronger among Turkish participants. In addition, the effects of *materialism* on *eudaimonic* and *HCT* were significantly stronger among Turkish participants, while the effect of *materialism* on the *snob effect* was stronger among Italian participants. No significant group differences were found for the remaining relationships. Accordingly, since significant differences emerged in four of the eight structural paths, H_7_ was partially supported. However, given the demographic differences between the two national samples, these between-country differences should not be attributed exclusively to cultural context, as differences in sample composition may also have contributed to the observed MGA results.

## 4. Discussion

In terms of cross-cultural differences, the country-specific analyses showed that the relationships observed in the pooled model were not uniformly reproduced within each national sample. In Türkiye, HCT and the snob effect were significantly associated with willingness to select coffeehouses, whereas in Italy, only the eudaimonic effect showed a significant association with willingness. Consistent with these country-specific patterns, the multi-group analysis showed that half of the structural relationships differed significantly between the two countries, and H7 was partially supported. Specifically, the relationship between eudaimonic motivations and customers’ willingness to select coffeehouses was stronger among Italian participants, whereas the relationship between HCT and willingness was stronger among Turkish participants. This pattern may reflect differences in the cultural positioning of coffee in the two contexts. Given that coffee is deeply embedded in daily routines in Italy [9], whereas coffeehouses in Türkiye are often associated with extended socialization and experiential sharing [14], these contextual differences may partly contribute to the observed variation in the strength of HCT and eudaimonic relationships. Similarly, the associations of materialism with HCT and eudaimonic motivations were stronger among Turkish participants, whereas its association with the snob effect was stronger among Italian participants. These differences may reflect different expressions of materialistic values across the two samples. However, such interpretations should be treated cautiously, as the observed differences cannot be attributed exclusively to culture. In contrast, no statistically significant between-country difference was detected for the paths involving the bandwagon effect. These non-significant differences should not be interpreted as evidence that the underlying mechanisms are equivalent across the two countries. Overall, the country-specific and multi-group results indicate that the structural relationships underlying coffeehouse selection are not uniform across the Turkish and Italian samples.

The IPMA findings further complement these results by showing that materialism had the highest importance (0.466) but below-average performance (33.45), placing it in the high-importance/low-performance quadrant. This finding highlights materialism as a priority area for managerial attention. However, this should not be interpreted as a need to increase consumers’ materialistic values; rather, it underscores the relevance of the symbolic and status-related meanings associated with coffeehouse selection. In contrast, eudaimonic motivation was positioned in the high-importance/high-performance quadrant, while HCT and the snob effect exhibited relatively lower importance but above-average performance.

### 4.1. Practical Implications

The research findings offer several practical implications for coffeehouse operators and marketing professionals. When examining the factors that shape consumers’ coffeehouse preferences, it becomes clear that not only the product or service offered, but also psychological, symbolic, and experiential motivations are relevant to coffeehouse selection. The existing literature highlights the critical function of the experiential process in coffeehouse selection, and the findings of this study support this view [10,11,12,65]. In this context, positioning coffeehouses solely as physical spaces for coffee consumption is insufficient; these spaces should be redefined as meaningful experiential areas where consumers can express their social identities, convey messages about their lifestyles, and develop a sense of belonging.

The research also found that M-CDS was positively associated with snob, bandwagon, HCT, and eudaimonic orientations. This finding indicates that consumers have different symbolic and emotional expectations and therefore supports the development of marketing approaches tailored to different consumer segments. For individuals with a strong snob orientation, exclusive products, limited-access offerings, premium atmospheres, and unique experiences may be particularly relevant, whereas for consumers susceptible to the bandwagon effect, social media visibility, perceived popularity, trend-based campaigns, and social approval mechanisms may be more salient. Moreover, the mediation findings showed significant indirect relationships between M-CDS and willingness to select coffeehouses through all four psychological orientations, with the largest specific indirect effect observed through the eudaimonic pathway. Therefore, rather than appealing to material values directly, managers could design experiences that address these psychological orientations, particularly by offering meaning-oriented elements such as authenticity, personal enrichment, and a sense of belonging. The IPMA further showed that M-CDS was positioned in the high-importance/low-performance quadrant, exhibiting the highest importance for willingness to select coffeehouses (0.466) but below-average performance (33.45). This positioning identifies M-CDS as a priority area for managerial attention. Rather than encouraging materialistic values directly, managers could address this priority by strengthening the symbolic and identity-expressive value of the coffeehouse experience, enabling customers to communicate their desired social image and lifestyle through their coffeehouse choices. However, although all four psychological orientations were significantly associated with willingness to select coffeehouses, their effect sizes were small (f^2^ = 0.018–0.045), indicating modest individual contributions to the outcome and suggesting that their managerial implications should not be overstated.

The findings are also consistent with the relevance of HCT and eudaimonic experiences in coffeehouse preference. Accordingly, businesses may benefit from moving beyond a product-centric perspective and adopting an approach that appeals to consumer emotions and imbues the entire experience process with meaning. Elements such as the atmosphere of the venue, aesthetic design, music selection, environmental arrangements that encourage social interaction, and experiential presentation methods may help strengthen the emotional connection individuals have with coffeehouses.

The cross-cultural differences observed in the study also have potential implications from a managerial perspective. The stronger relationship between HCT and willingness to select coffeehouses among Turkish participants suggests that marketing strategies focused on pleasure, sensory satisfaction, and vivid experiences may be particularly relevant in the Turkish context. On the other hand, the stronger relationship between eudaimonic motivation and willingness among Italian participants suggests that meaningful, authentic, and lifestyle-integrated experiences, including approaches associated with personal meaning and well-being, may be particularly relevant in the Italian context. However, these managerial interpretations should be treated cautiously because the observed between-country differences cannot be attributed exclusively to culture. Therefore, coffeehouse brands operating internationally may benefit from adapting their experience designs to the characteristics of different markets rather than assuming that a uniform strategy will be equally relevant across contexts.

### 4.2. Theoretical Implications

The research findings offer several theoretical contributions to the literature on consumer behavior, experiential consumption, and coffeehouse consumption. First, this study contributes to the literature by organizing coffeehouse selection within a value–motivation–intention framework. Within this framework, M-CDS represents the underlying value orientation, while HCT, eudaimonic, snob, and bandwagon orientations represent distinct psychological and motivational pathways associated with customers’ willingness to select coffeehouses. In this way, the study demonstrates the usefulness of examining coffeehouse selection as a process in which an underlying consumer value is associated with behavioral intention through more proximal consumption-related orientations. The significant specific indirect effects through all four psychological orientations provide empirical support for these indirect relationships. Thus, the findings extend the value–motivation–intention logic to the coffeehouse context by showing how a contextualized materialistic value orientation may be linked to behavioral intention through multiple social and experiential pathways.

Another theoretical contribution of the study concerns how coffeehouses are considered beyond their role as functional spaces for consumption. In this research, coffeehouses are conceptualized as multi-layered consumption spaces where social visibility, belonging, status display, and experiential meaning production occur simultaneously. In the literature, coffeehouses are largely examined through dimensions such as service quality, customer satisfaction, and atmosphere [3,8]. However, this research focuses on the psychological and symbolic motivations underlying consumers’ coffeehouse preferences, making the social and experiential dimensions of these choices more visible. Furthermore, the study examines HCT, eudaimonic, snob, and bandwagon tendencies together within a single model; consumers’ psychological and symbolic motivational structures are evaluated from a multidimensional perspective. Especially with the inclusion of eudaimonic processes in the analysis, the study goes beyond existing research, suggesting that meaning, personal fulfillment, and other eudaimonic aspects may also be relevant to coffeehouse selection. The positive relationship between the snob effect and willingness to select coffeehouses further indicates that distinction-oriented motives can also contribute to coffeehouse selection, although the magnitude of this relationship was modest. At the same time, M-CDS explained substantially more variance in the bandwagon effect (R^2^ = 0.522) than in the snob effect (R^2^ = 0.068), suggesting that its explanatory role is considerably stronger for conformity-oriented than distinction-oriented consumption tendencies.

One of the most striking findings of the research is the significant relationship between M-CDS and eudaimonic motivation. In the traditional literature, materialism is mostly associated with superficial consumption practices, the drive for possession, and hedonistic processes [31,32]. However, the positive association observed between M-CDS and eudaimonic motivation suggests that materialistic value orientations may also coexist with consumers’ pursuit of meaning, personal fulfillment, and other eudaimonic aspects in experiential and symbolic consumption contexts. This finding is particularly relevant because previous research on materialism and coffee consumption has primarily examined snob, bandwagon, and hedonic consumption tendencies [17], whereas the present study extends this line of research by incorporating an eudaimonic pathway. In recent years, coffeehouses have transformed into spaces symbolizing modern lifestyle, social visibility, and experiential sharing [14]. Accordingly, the observed relationship suggests that the symbolic and experiential meanings attached to coffeehouse consumption may provide a context in which materialistic value orientations are associated with eudaimonic as well as social and hedonic motivations. This interpretation is further supported by the IPMA, in which M-CDS exhibited the highest importance for willingness to select coffeehouses, despite its comparatively low performance. This finding reinforces the theoretical relevance of contextualized materialism within the value–motivation–intention framework, suggesting that its role in coffeehouse selection extends beyond its individual structural relationships with specific motivational orientations.

Finally, the findings based on a comparison between Türkiye and Italy suggest that the value–motivation–intention relationships examined in this study are not uniform across the two national samples. The relationship between HCT and willingness to select coffeehouses was stronger among Turkish participants, whereas the relationship between eudaimonic motivation and willingness was stronger among Italian participants. These findings indicate that the social and experiential pathways associated with coffeehouse selection may vary across different national contexts. However, these differences should be interpreted cautiously, as they cannot be attributed exclusively to culture, particularly given the demographic differences between the two samples.

### 4.3. Limitations and Future Studies

Since these findings stem specifically from Turkish and Italian samples, caution is warranted in extending them to other regions or subgroups. The convenience sampling procedure also limits the representativeness of both national samples. In particular, the samples differed substantially in age, gender, and education, and no respondents aged 18–24 years were represented in the Turkish sample. These demographic differences may partly account for the between-country differences observed in the MGA, limiting their interpretation as purely cultural effects. Future cross-cultural research should therefore employ demographically matched or quota-based samples. In addition, the cross-sectional and self-reported nature of the data limits causal inference; thus, the observed relationships should be interpreted as associations rather than causal effects. A further limitation concerns the willingness to select coffeehouses construct, which was measured with only two items and may therefore have limited internal consistency. Another limitation concerns discriminant validity. Although the Fornell–Larcker criterion was satisfied, the HTMT assessment revealed that several construct pairs exceeded the commonly recommended thresholds, indicating substantial empirical overlap among some of the theoretically distinct constructs. Accordingly, relationships involving these constructs should be interpreted with caution, and future research should further examine their empirical distinctiveness using alternative samples and measurement specifications. Future studies could address these limitations by using more comprehensive measures, longitudinal or experimental designs, additional cultural settings, and testing the model’s predictive validity in new samples.

## 5. Conclusions

This study contributes to the literature in three ways. Theoretically, it organizes coffeehouse selection within a value–motivation–intention framework by conceptualizing M-CDS as the underlying value orientation and snob, bandwagon, HCT, and eudaimonic orientations as psychological and motivational pathways associated with behavioral intention. It further extends prior work on materialism and coffee consumption [17] by adding the eudaimonic pathway and examining the full structural model across two culturally distinct samples. Importantly, the country-specific analyses showed that the relationships observed in the pooled model were not uniformly reproduced within each national sample. In Türkiye, HCT and the snob effect were significantly associated with willingness to select coffeehouses, whereas in Italy, only the eudaimonic effect showed a significant association with willingness. These findings underscore the importance of examining structural relationships within each cultural context rather than relying exclusively on aggregate estimates. Practically, the findings indicate that coffeehouse operators should move beyond a product-centric positioning toward experience designs that address distinct psychological orientations—status-driven, conformity-driven, pleasure-driven, and meaning-driven—while recognizing that their relevance may vary across national contexts. The IPMA further highlights M-CDS as a key managerial priority due to its high importance but comparatively low performance, underscoring the potential value of strengthening the symbolic and identity-expressive aspects of the coffeehouse experience rather than directly promoting materialistic values. Methodologically, the study’s analytical workflow—evaluating the model separately within each national sample, establishing measurement invariance via MICOM before multi-group analysis, and assessing out-of-sample predictive validity via PLSpredict and CVPAT—provides a comprehensive approach to examining cross-cultural PLS-SEM models. Future research should address the limitations noted above, particularly by incorporating demographic controls and employing longitudinal, multi-wave, or experimental designs to provide stronger evidence regarding the relationships identified in this study.

## Figures and Tables

**Table 1:** Participant profile.

Categories		Türkiye		Italy		Total	
		N	%	N	%	N	%
**Age (yrs)**	18–24	-	-	23	7.3	37	6.2
	25–34	148	51.7	62	19.7	171	28.5
	35–44	78	27.3	45	14.3	140	23.3
	45–54	42	14.7	68	21.6	87	14.5
	55–64	13	4.5	80	25.4	81	13.5
	65 and older	5	1.7	37	11.7	85	14.1
**Gender**	Female	183	64.0	95	30.2	278	46.3
	Male	98	34.3	218	69.2	316	52.6
	Prefer not to say	5	1.7	2	0.6	7	1.2
**Marital status**	Married	75	26.2	166	52.7	241	40.1
	Single	211	73.8	149	47.3	360	59.9
**Education**	Less than high school	1	0.3	11	3.5	12	2.0
	High school	76	26.6	77	24.4	153	25.5
	Technical or Associate Degree	54	18.9	15	4.8	69	11.5
	Bachelor’s Degree	89	31.1	55	17.5	144	24.0
	Master’s Degree	36	12.6	118	37.5	154	25.6
	Doctorate (PhD)	30	10.5	39	12.4	69	11.5
**Self-Perceived Income Level**	Very low	26	9.1	4	1.3	30	5.0
	Low	53	18.5	34	10.8	87	14.5
	Middle	173	60.5	232	73.7	405	67.4
	High	30	10.5	39	12.4	69	11.5
	Very high	4	1.4	6	1.9	10	1.7
**Frequency of Coffeehouse Visits**	Rarely	106	37.1	55	17.5	55	9.2
	Sometimes (1–3/month)	90	31.5	95	30.2	161	26.8
	1 to 2 times/week	53	18.5	86	27.3	185	30.8
	3 to 4 times/week	17	5.9	44	14.0	139	23.1
	5 or more times/week	20	7.0	35	11.1	61	10.1

Source. Created by the authors.

**Table 2:** Reliability and validity scores of constructs.

Construct	λ	OuterVIF	rho_a	rho_c	AVE
**Materialism—coffeehouse as defining success (M-CDS) (α = 0.630)**			**0.638**	**0.803**	**0.577**
The cafe I visit says a lot about how well I’m doing in life	0.825	1.402			
I like to visit coffeehouses that impress people	0.690	1.168			
I admire people who visit coffeehouses	0.758	1.291			
**Bandwagon effect (BWagon) (α = 0.807)**			**0.810**	**0.866**	**0.563**
If I want to be like someone, I often try to visit the same coffeehouses that they also visit	0.754	1.757			
I often identify with other people by going to the same coffeehouses they go to	0.761	1.646			
If other people can see me go to a coffeehouse, I often choose the place they expect me to go	0.733	1.552			
I achieve a sense of belonging by visiting the same coffeehouses that others visit	0.780	1.645			
I like to know that coffeehouses make a good impression on others	0.723	1.375			
**Snob effect (Snob) (α = 0.814)**			**0.833**	**0.876**	**0.639**
I often try to avoid coffeehouses that I know are frequently visited by the general population	0.786	1.752			
When a coffeehouse I usually visit becomes popular among the general population, I begin to visit less	0.832	1.628			
The more commonplace a coffeehouse is among the general population, the less interested I am in visiting it	0.785	1.668			
As a rule, I dislike coffeehouses that are customarily visited by everyone	0.793	1.673			
**Hedonic consumption tendency (HCT) (α = 0.847)**			**0.848**	**0.897**	**0.685**
Visiting coffeehouses offers new experiences	0.823	1.889			
Visiting coffeehouses satisfies my sense of curiosity	0.829	1.846			
I like to visit coffeehouses for the novelty of the experience	0.838	2.017			
I feel like I’m exploring new worlds when I visit coffeehouses	0.821	1.819			
**Eudaimonic effect (Eudaimonic) (α = 0.837)**			**0.838**	**0.891**	**0.672**
The coffeehouses experience helped me feel a sense of accomplishment and fulfillment	0.806	1.757			
The coffeehouses experience stimulated my reflection	0.800	1.721			
The coffeehouses experience made a lot of sense to me	0.831	1.911			
The coffeehouses experience helped me develop a positive attitude toward myself	0.841	1.946			
**Customers’ willingness to select coffeehouses (Willingness) (α = 0.476)**			**0.484**	**0.791**	**0.655**
I would prefer coffeehouses over other ways	0.843	1.108			
I would select coffeehouses to drink coffee	0.774	1.108			

Source. Created by authors.

**Table 3:** Fornell–Larcker criterion.

No	Constructs	1	2	3	4	5	6
**1**	Bandwagon effect	**0.751**					
**2**	Eudaimonic effect	0.622	**0.820**				
**3**	Hedonic consumption tendency	0.479	0.764	**0.828**			
**4**	Materialism	0.723	0.715	0.651	**0.760**		
**5**	Snob effect	0.246	0.203	0.204	0.260	**0.799**	
**6**	Customers’ willingness to select coffeehouses	0.442	0.551	0.506	0.506	0.248	**0.809**

Note. Numbers in bold are AVE square root results. Source. Created by the authors.

**Table 4:** Structural equation model results.

Hypotheses		β	{$\bar{x}$}	SD	t-Statistics	*p*-Value	InnerVIF	f^2^	Results
**H_1a_**	Materialism -> Snob effect	0.260	0.263	0.042	6.192	0.000 ***	1.000	0.073	Supported
**H_1b_**	Materialism -> Bandwagon	0.723	0.723	0.023	30.991	0.000 ***	1.000	1.094	Supported
**H_1c_**	Materialism -> HCT	0.651	0.652	0.025	26.562	0.000 ***	1.000	0.736	Supported
**H_1d_**	Materialism -> Eudaimonic effect	0.715	0.715	0.021	33.329	0.000 ***	1.000	1.047	Supported
**H_2_**	Snob effect -> Willingness	0.115	0.116	0.037	3.114	0.002 **	1.075	0.019	Supported
**H_3_**	Bandwagon -> Willingness	0.138	0.137	0.045	3.085	0.002 **	1.673	0.018	Supported
**H_4_**	HCT -> Willingness	0.189	0.191	0.061	3.088	0.002 **	2.420	0.023	Supported
**H_5_**	Eudaimonic effect -> Willingness	0.298	0.297	0.069	4.298	0.000 ***	3.025	0.045	Supported

Source. Created by authors, *p* ≤ 0.001 ***, *p* ≤ 0.05 **; Willingness = Customers’ willingness to select coffeehouses, HCT = Hedonic consumption tendency, $\bar{x}$ = mean, SD = Standard deviation.

**Table 5:** PLS Predict and CVPAT results.

Constructs	Items	Q^2^_predict_	PLS-SEM_MAE	LM_MAE	IA Average Loss Difference (*p*-Value)	LM Average Loss Difference (*p*-Value)
**Bandwagon**	Bwagon1	0.227	0.720	0.718	−0.400 (0.000)	0.002 (0.646)
	Bwagon2	0.297	0.742	0.748		
	Bwagon3	0.241	0.747	0.757		
	Bwagon4	0.292	0.769	0.776		
	Bwagon5	0.370	0.845	0.811		
**Eudaimonic**	Eudaimonic1	0.378	0.805	0.787	−0.530 (0.000)	0.001 (0.846)
	Eudaimonic2	0.316	0.844	0.848		
	Eudaimonic3	0.320	0.807	0.808		
	Eudaimonic4	0.350	0.816	0.821		
**HCT**	Hedonic1	0.282	0.882	0.874	−0.459 (0.000)	0.011 (0.172)
	Hedonic2	0.321	0.843	0.843		
	Hedonic3	0.236	0.917	0.899		
	Hedonic4	0.310	0.864	0.861		
**Snob**	Snob1	0.021	1.077	1.074	−0.065 (0.007)	0.008 (0.545)
	Snob2	0.059	1.091	1.079		
	Snob3	0.031	1.000	0.994		
	Snob4	0.045	1.074	1.068		
**Willingness**	Willingness1	0.182	0.934	0.932	−0.270 (0.000)	0.022 (0.113)
	Willingness2	0.148	1.013	0.979		
**Overall**					−0.355 (0.000)	0.007 (0.065)

Source. Created by authors, Willingness = Customers’ willingness to select coffeehouses, HCT = Hedonic consumption tendency.

**Table 6:** Mediation effect results.

Hypotheses		β	{$\bar{x}$}	SD	t-Statistics	*p*-Values
**H_6a_**	Materialism -> Bandwagon -> Willingness	0.100	0.099	0.033	3.048	0.002 **
**H_6b_**	Materialism -> HCT -> Willingness	0.123	0.125	0.041	3.022	0.003 **
**H_6c_**	Materialism -> Snob -> Willingness	0.030	0.030	0.011	2.701	0.007 **
**H_6d_**	Materialism -> Eudaimonic -> Willingness	0.213	0.213	0.050	4.228	0.000 ***

Source. Created by authors, *p* ≤ 0.001 ***, *p* ≤ 0.05 **; Willingness = Customers’ willingness to select coffeehouses.

**Table 7:** MGA results.

Construct	β (Italy)	β (Türkiye)	Difference (Italy—Türkiye)	t-Statistics (Italy—Türkiye)	(Italy vs. Türkiye) *p*-Value	Significance Difference
Bandwagon effect -> Willingness	0.121	0.080	0.041	0.443	0.658	No
Eudaimonic effect -> Willingness	0.440	0.157	0.283	2.057	0.040 **	Yes
HCT -> Willingness	0.091	0.342	−0.251	2.060	0.040 **	Yes
Materialism -> Bandwagon effect	0.682	0.734	−0.052	1.086	0.278	No
Materialism -> Eudaimonic effect	0.638	0.781	−0.143	3.283	0.001 ***	Yes
Materialism -> HCT	0.590	0.712	−0.122	2.462	0.014 **	Yes
Materialism -> Snob effect	0.345	0.127	0.218	2.516	0.012 **	Yes
Snob effect -> Willingness	0.026	0.157	−0.131	1.741	0.082	No

Source. Created by authors, *p* ≤ 0.001 ***, *p* ≤ 0.05 **, Willingness = Customers’ willingness to select coffeehouses, HCT = Hedonic consumption tendency.

## Data Availability

The data supporting the findings of this study are available from the corresponding author upon reasonable request.

## Supplementary Materials

The following supporting information can be downloaded at: https://www.mdpi.com/article/10.3390/foods15203585/s1, Table S1: Checklist for Reporting Results of Internet E-Surveys (CHERRIES); Figure S1: Structural model equation model scores for Italy; Source. Created by authors; Figure S2: Structural model equation model scores for Türkiye; Source. Created by authors.
