# Supplementary material for: Coffeehouses as Symbolic Spaces: Materialism and Experiential Motivations in Coffeehouse Selection
Source: Foods. 2026 Oct 9;15(20):3585. doi: 10.3390/foods15203585 (PMC13703210; doi:10.3390/foods15203585)
Supplement: Supplementary file 1 [file foods-15-03585-s001.zip › foods-4549227-supplementary.pdf]

## SUPPLEMENTARY MATERIALS

**Supplementary Table S1:** Checklist for Reporting Results of Internet E-Surveys (CHERRIES)

| Item category | Checklist item | Reporting in the present study |
|---|---|---|
| **Design** | Describe survey design | A cross-sectional online survey was conducted among individuals aged 18 years or older who patronized coffeehouses in Türkiye or Italy. Convenience sampling was used. |
| **IRB approval and informed consent** | IRB approval | The study received approval from the relevant institutional ethics committee. The approval number and date are reported in the Ethics Statement of the manuscript. |
| | Informed consent | Before beginning the questionnaire, participants were informed about the purpose of the study, the voluntary nature of participation, and their right to discontinue participation. Written informed consent was obtained before proceeding to the survey. |
| | Data protection | No directly identifying personal information, such as names, telephone numbers, or e-mail addresses, was collected. |
| **Development and pre-testing** | Development and testing | The original English measures were translated into Turkish and Italian using a translation–back-translation procedure. Academic specialists in tourism and consumer behavior reviewed the wording and cultural appropriateness. The questionnaire was pilot-tested with 30 participants, who were not included in the final analytical sample, and minor linguistic refinements were made before data collection. |
| **Recruitment process and sample access** | Open versus closed survey | The survey was open; respondents who had access to the survey link could enter the questionnaire without a password or individualized invitation link. Eligibility criteria were assessed at the beginning of the questionnaire. |
| | Contact mode | Potential participants were approached through both online and face-to-face channels in Türkiye and Italy. |
| | Advertising/ recruitment | Participants were recruited through social media, WhatsApp, and face-to-face contact. The same recruitment approaches were used in both countries. |
| **Survey administration** | Web/e-mail | The questionnaires were administered online using QualtricsXM. Separate survey links and language versions were used for Türkiye and Italy, and data were collected separately in the two countries. |
| | Context | The survey investigated coffeehouse-related consumer perceptions and willingness to select coffeehouses among consumers in Türkiye and Italy. |
| | Mandatory/voluntary | Participation in the study was entirely voluntary. Once respondents chose to participate and proceeded with the questionnaire, responses to survey items were required. |
| | Incentives | No monetary or non-monetary incentives were offered for participation. |
| | Time/date | Data were collected between December 25, 2022 and June 6, 2023. Data collection was concluded when 601 completed responses had been obtained (Türkiye: n = 286; Italy: n = 315). |
| | Randomization | Items and construct blocks were presented in the same order to all respondents; no item randomization was applied. |
| | Adaptive questioning | Adaptive questioning was not used for the substantive questionnaire. Eligibility screening at the beginning of the survey determined whether respondents could proceed. |
| | Number of items | The questionnaire contained 28 items/questions, including demographic questions. |
| | Number of screens/pages | The exact number of survey screens/pages was not retained and is therefore not available for reporting. |
| | Completeness check | Responses to questionnaire items were required once participants entered the survey. Incomplete questionnaires were excluded from the analytical dataset. |
| | Review step | Respondents were able to return to previous pages and review or change their responses using the Back function. |
| **Response metrics** | Unique site visitor | The survey was distributed through direct survey links rather than through a host website for which unique visitor statistics were available. Therefore, this metric was not available. |
| | View rate | Not available because the number of individuals exposed to or viewing the survey links was not recorded. |
| | Participation rate | Not calculable because the total number of unique individuals who accessed the first survey page was not retained. |

| | | |
|---|---|---|
| | Completion rate | Not calculable because the total number of individuals who initiated the questionnaire was not retained. The final analytical dataset contained 601 completed questionnaires. |
| **Preventing multiple entries** | Cookies / duplicate prevention | Qualtrics$^{XM}$ *Prevent Multiple Submissions* function was used to reduce the possibility of multiple submissions by the same respondent. |
| | IP check | IP addresses were checked for potential duplicate responses. No completed responses were excluded on this basis. |
| | Log-file analysis | No additional log-file analysis was conducted beyond the duplicate-prevention and IP-based checks described above. |
| **Analysis** | Handling of incomplete questionnaires | Partial/incomplete questionnaires were excluded from the analytical dataset; only completed questionnaires were included in the final sample. |
| | Atypical timestamps/speeding | Survey completion times were examined to identify unusually rapid responses. No predetermined minimum completion-time threshold was imposed, and no completed responses were excluded on this basis. |
| | Straight-lining | Response patterns were examined for straight-lining. No problematic straight-lining cases were identified; therefore, no completed responses were excluded on this basis. |
| **Additional administration information** | Eligibility screening | Respondents were required to be aged 18 years or older and to patronize coffeehouses. Eligibility was assessed at the beginning of the questionnaire, and individuals who did not meet the eligibility criteria could not proceed with the survey. |
| | Survey access | The questionnaire could be accessed using computers, tablets, and mobile devices. |
| | Country administration | Data collection was conducted separately in Türkiye and Italy using separate Qualtrics$^{XM}$ links and the corresponding Turkish and Italian questionnaire versions. |

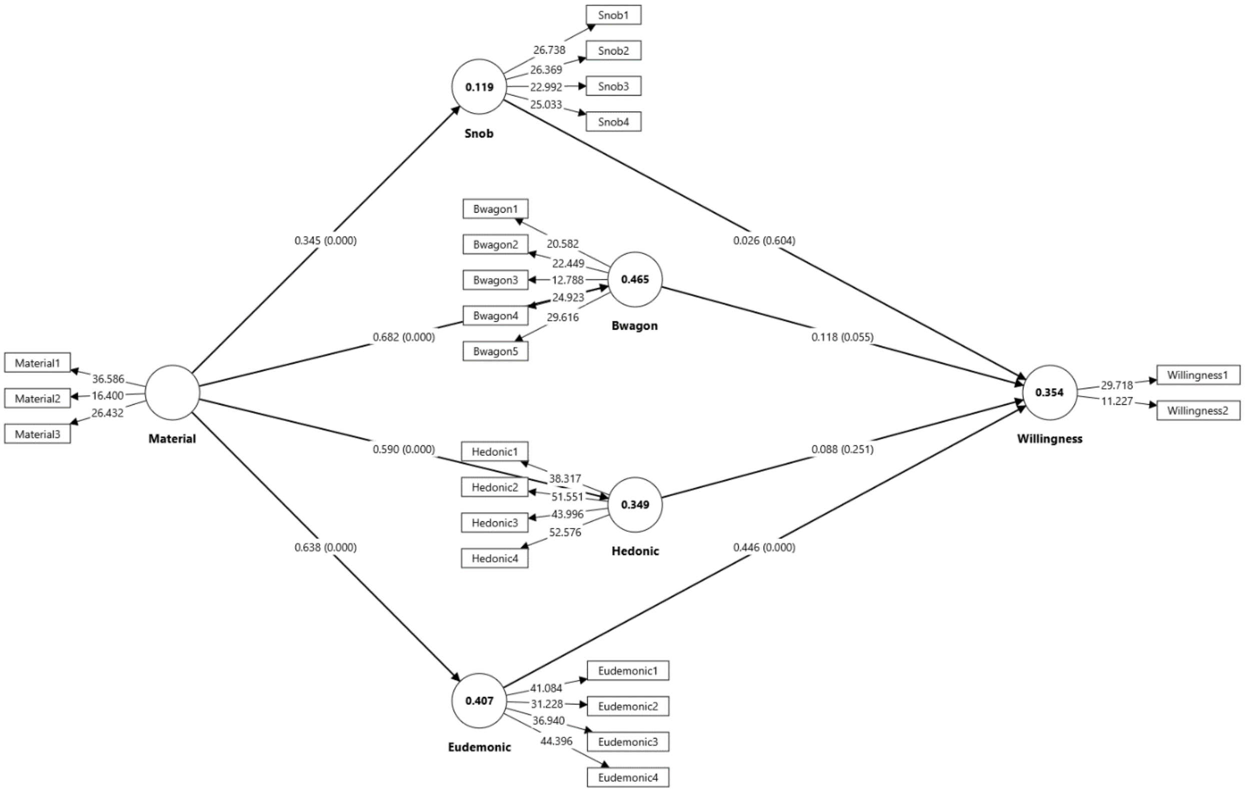


**Supplementary Figure S1:** Structural model equation model scores for Italy

*Source.* Created by authors.

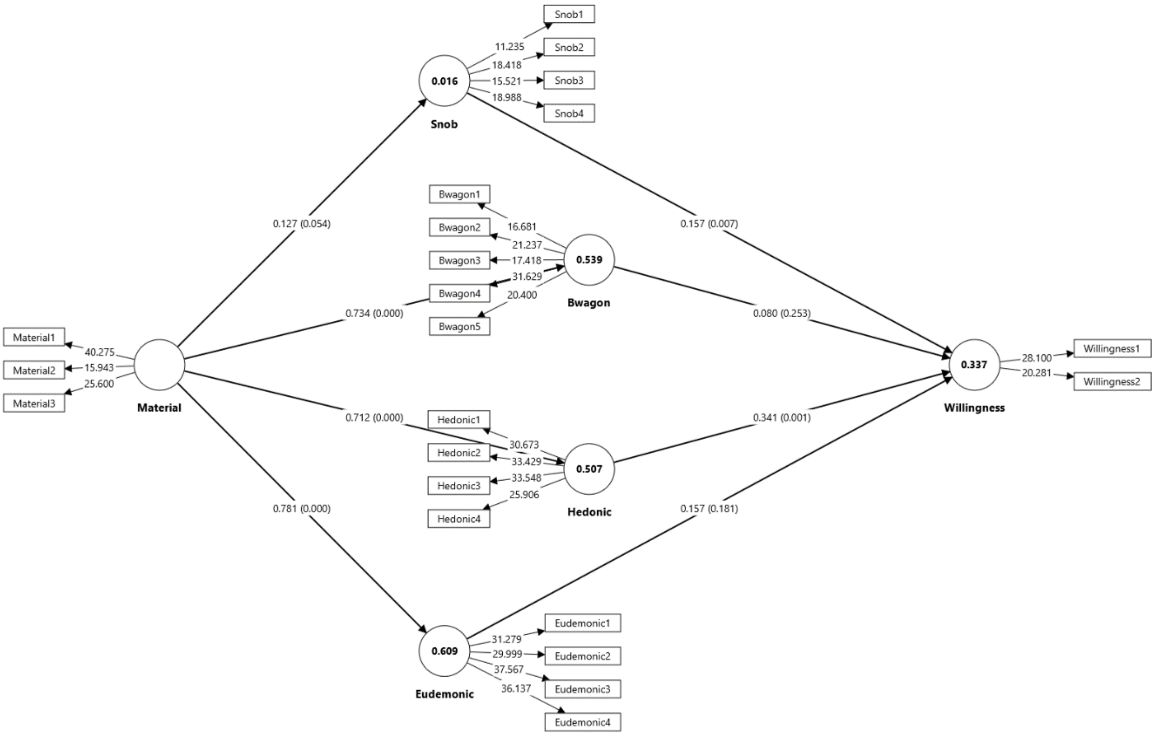


**Supplementary Figure S2:** Structural model equation model scores for Türkiye

*Source.* Created by authors.
